# Risk factors for subsequent thyroid neoplasm development in childhood cancer survivors

**DOI:** 10.1530/ERC-26-0210

**Published:** 2026-10-23

**Authors:** Dana Nováková, Karel Švojgr, Rami Katra, Tereza Bartůňková, Petr Vlček, Anna Kindlová, Michaela Jirkovská, Sarah Blagodárná, Tomáš Eckschlager, Lucie Šrámková, Jarmila Kruseová

**Affiliations:** ^1^ Department of Nuclear Medicine and Endocrinology, 2nd Faculty of Medicine, Charles University and Motol and Homolka University Hospital, Prague, Czech Republic; ^2^ Department of Pediatric Hematology and Oncology, 2nd Faculty of Medicine, Charles University and Motol and Homolka University Hospital, Prague, Czech Republic; ^3^ Department of Ear, Nose and Throat, 2nd Faculty of Medicine, Charles University and Motol and Homolka University Hospital, Prague, Czech Republic; ^4^ Department of Oncology, 2nd Faculty of Medicine, Charles University and Motol and Homolka University Hospital, Prague, Czech Republic

**Keywords:** childhood cancer survivors, subsequent thyroid carcinoma, subsequent thyroid adenoma, risk factors

## Abstract

Subsequent thyroid carcinomas (STCs) are common after childhood cancer treatment and have well-described risk factors, whereas information on subsequent thyroid adenomas (STAs) remains limited. This study compared treatment-related risk factors for STCs and STAs, assessed their associations with other subsequent neoplasms, and evaluated the incidence of STCs relative to that in the age-matched national general population. The study cohort included 4,348 childhood cancer survivors treated between 1975 and 2020; 48% were female, 23% had lymphomas, and 77% had solid tumors. STCs occurred in 44 survivors (59% female), and STAs occurred in 23 (65% female). Compared with the national general population, the risk of STCs was 7.6-fold higher in females and 23.6-fold higher in males. The median age at diagnosis was lower for STCs (25.8 vs 29.8 years). Hodgkin lymphoma was the most frequent primary cancer in both groups (48% vs 57%). The median age at first cancer diagnosis was similar between the groups (9.8 vs 9.4 years). Neck radiotherapy was a major risk factor in both groups (59% vs 83%). Additional subsequent malignant neoplasms occurred in 14% of patients with STCs and 13% of patients with STAs, whereas subsequent benign neoplasms occurred in 20% and 26%, respectively. The incidence of subsequent benign neoplasms was higher in patients with STCs than in patients with other first subsequent malignant neoplasms (*P* < 0.05). STCs predominated over STAs (66%). Shared risk factors included neck radiotherapy and Hodgkin lymphoma. STCs were diagnosed at a younger age. The frequent occurrence of additional subsequent neoplasms highlights the need for long-term surveillance.

## Introduction

The development of subsequent malignant neoplasms (SMNs) is one of the most serious life-threatening late effects of childhood cancer treatment (1, 2). Subsequent thyroid carcinoma (STC) is among the most common malignancies in childhood cancer survivors (2, 3, 4, 5, 6, 7). The most significant risk factor for thyroid cancer is radiation exposure (8). It is well established that radiation exposure to the neck region – whether resulting from therapeutic irradiation for tumors, nuclear power plant accidents, or atomic bomb explosions – significantly increases the risk of developing thyroid cancer (9, 10, 11, 12, 13, 14). In contrast, very few studies have focused on risk factors for developing subsequent thyroid adenomas (STAs) in childhood cancer survivors (15, 16, 17). According to the KiKme study, 24% of survivors were diagnosed with thyroid disorders other than cancer, including benign nodules and STAs (16). Radiotherapy has been shown to be associated with a similar risk of developing STAs and STCs (18, 19, 20). In addition to radiotherapy and prior chemotherapy, female sex and younger age at primary cancer diagnosis have also been associated with a higher risk of thyroid pathology, including STCs and STAs (7, 10, 11, 15).

With advancing age, childhood cancer survivors may develop multiple subsequent neoplasms (21, 22, 23, 24). The occurrence of these subsequent neoplasms in survivors is likely influenced not only by well-established hereditary cancer predisposition syndromes but also by broader genetic susceptibility factors. Supporting this hypothesis, polygenic risk scores have been associated with the risk of developing STCs and other subsequent neoplasms (25). For example, the highest incidence of STCs has been reported among survivors with elevated polygenic risk scores who were exposed to neck radiotherapy doses of 10 to <30 Gy (25). Data from the Childhood Cancer Survivor Study and the Dutch Childhood Oncology Group – Long-Term Effects After Childhood Cancer Study also indicated that subsequent benign neoplasms (SBNs) may serve as effective risk markers for SMN development (22). This association may reflect shared etiological mechanisms underlying the development of both SBNs and SMNs (22, 23, 24). However, limited information is available on whether survivors with STCs or STAs are subsequently at increased risk of developing other SMNs or SBNs, except for individuals with known genetic cancer predisposition syndromes (26). Therefore, we analyzed our cohort data.

The primary aim of this study was to compare the incidence of STCs and STAs, identify treatment-related risk factors associated with their development, and compare the incidence of STCs in childhood cancer survivors with the age- and sex-specific incidence in the national general population. The secondary objective was to assess whether the occurrence of subsequent thyroid neoplasms is associated with an increased risk of developing additional subsequent neoplasms.

## Materials and methods

The study cohort consisted of 4,348 childhood cancer survivors who were regularly followed up at the Long-Term Follow-Up Clinic Prague (LCP). All survivors had been treated at the Department of Pediatric Hematology and Oncology, were <19 years old at the time of their first cancer diagnosis, and were in complete remission at least 5 years after the end of therapy. The cohort included patients treated for lymphomas (23%) and solid tumors (77%), including CNS tumors. A summary of the whole cohort definition is provided in Fig. 1. Within this cohort, all patients with cervical lymph node involvement underwent regular neck ultrasound examinations during the first 5 years after completion of therapy, in accordance with diagnosis-specific follow-up recommendations. In addition, all survivors who had received radiotherapy involving the thyroid region or treatment with radioactive iodine (^131^I-MIBG) underwent long-term thyroid surveillance initiated 5 years after radiotherapy. The frequency of subsequent follow-up examinations was determined by the endocrinologist based on thyroid ultrasound findings. Only patients with thyroid neoplasms confirmed by histopathological examination following total thyroidectomy were included in the study. All patients were followed up regularly at our institution. Patients with genetic syndromes were excluded.

**Figure 1:**
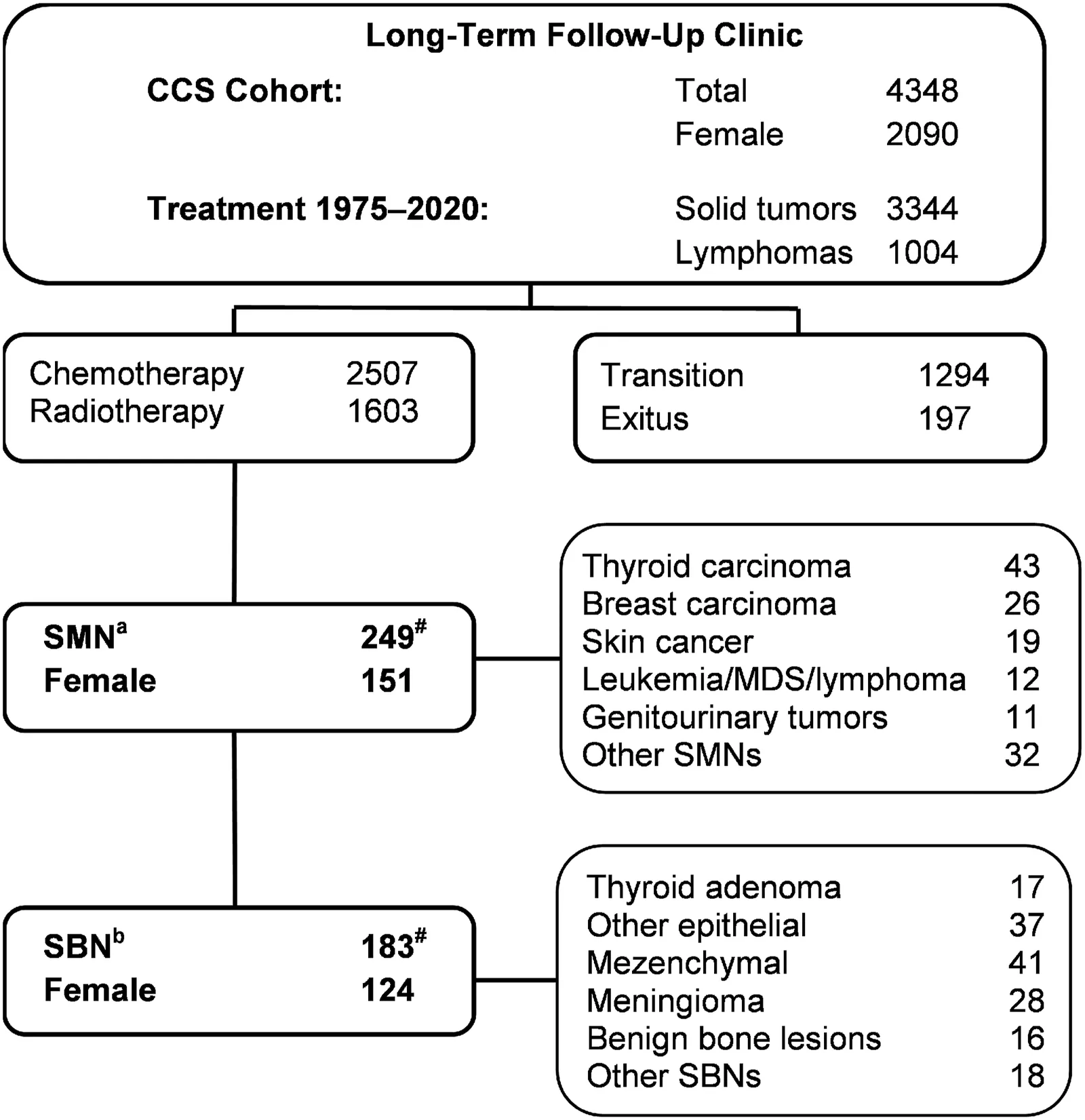
Summary of the cohort definition. SMNs^a^, subsequent malignant neoplasms; SBNs^b^, subsequent benign neoplasms. ^#^including patients with genetic syndromes and patients who died ≤5 years after the diagnosis of the first subsequent neoplasm. Transition* – patients ≥40 years of age or patients <40 years of age who transferred to another facility at their own request.

The expected number of thyroid cancers was estimated by applying age-, sex-, and calendar year-specific incidence rates from the national cancer registry (accessed July 30, 2026) to the cohort-specific person-years at risk. As population incidence data were unavailable for 1975 and 1976, incidence rates were approximated using values from 1977. For 2025, incidence rates were approximated using the most recent available data from 2024. Survivors were routinely followed until 40 years of age; therefore, only person-years accrued before age 41 were included in the analysis.

To evaluate the incidence of other SMNs and SBNs, we used our subsequent neoplasms cohort. SBNs were always histologically confirmed. To evaluate the risk of developing multiple subsequent neoplasms after the first SMN or SBN, we excluded patients who died within 5 years after the diagnosis of the first subsequent neoplasm and those with confirmed genetic syndromes. We compared the incidence of additional tumors in patients with STCs and STAs with that of multiple subsequent neoplasms at other anatomical sites.

The primary objective of this study was to assess the incidence of STCs and STAs, evaluate treatment-related risk factors associated with prior cancer therapy, and compare the incidence of STCs in childhood cancer survivors with the age- and sex-specific incidence in the national general population. The secondary objective was to determine the incidence of other subsequent neoplasms among patients diagnosed with STCs or STAs.

### Statistical analysis

Statistical analyses were performed using R version 4.5.1 (R Foundation for Statistical Computing, Vienna, Austria; 2025; available at https://www.R-project.org/). Categorical variables were summarized as frequencies and percentages. Differences between groups were assessed using Pearson’s chi-square test or Fisher’s exact test, as appropriate. All tests were two-sided, and *P* values < 0.05 were considered statistically significant. To compare the incidence of STCs with that in the age-matched national general population, the standardized incidence ratio (SIR) was calculated as the ratio of the observed to the expected number of cases. The expected number of cases was derived from age-specific incidence rates in the national general population. The 95% CI for the observed number of cases was estimated using exact Poisson confidence intervals.

## Results

1a) Overall, 44 survivors developed STCs, corresponding to a cumulative incidence of 1.0%; 26 (59%) were female. STAs were diagnosed in 23 survivors (cumulative incidence, 0.5%), of whom 15 (65%) were female. The proportion of women was 61.2% among patients with subsequent thyroid neoplasms, compared with 48.0% in the primary cancer cohort and 63.7% among patients with other subsequent neoplasms. The comparison with primary cancer was statistically significant, though we cannot rule out that the increase is almost negligible (*χ²* = 4.04, *P* = 0.04; OR = 1.70, 95% CI: 1.01–2.91), while the comparison against other subsequent neoplasms was not significant (*χ²* = 0.06, *P* = 0.80; OR = 0.90, 95% CI: 0.52–1.60).

The median age at STC diagnosis was 25.8 years (interquartile range, IQR: 18.2–32.6), compared with 29.8 years (IQR: 21.4–35.7) at STA diagnosis. Papillary thyroid carcinoma was the most frequent histopathological type of STC, occurring in 38 patients (86%), whereas follicular adenoma was the most frequent histopathological type of STA, occurring in 12 patients (52%). At the time of STC diagnosis, 19 patients (43%) had stage T1a carcinoma, two patients (5%) had T1b, seven patients (16%) had T2, nine patients (20%) had T3, and seven patients (16%) had T4 tumors. Lymph node metastases were present in 16 patients (36%), and distant metastases were identified in four patients (9%). A detailed comparison and characterization of STCs and STAs are presented in Table 1.

**Table 1:** Comparative summary of STC and STA.

Thyroid SN	Histopathological analysis	F/M	Median age at first cancer diagnosis	Median time to the development of thyroid SN	Median age at thyroid SN
STC^a^	Papillary carcinoma 38 pts. (86%) Follicular carcinoma 5 pts. (11%) Oncocytic carcinoma 1 pt. (2%)	All patients (44)	9.8 years	15.8 years	25.8 years
			IQR^●^ 4.1–14.6	IQR^●^ 11.1–20.1	IQR^●^ 18.2–32.6
		F 26 (59%)	12.4 years	16.1 years	26.4 years
			IQR^●^ 3.1–14.8	IQR^●^ 12.1–19.1	IQR^●^ 19.3–32.9
		M 18 (41%)	9.1 years	14.8 years	23.3 years
			IQR^●^ 4.4–12.8	IQR^●^ 10.6–22.9	IQR^●^ 17.6–31.7
STA^b^	Follicular adenoma 12 pts. (52%) Oncocytic adenoma 7 pts. (30%) Unspecified adenoma 4 pts. (17%)	All patients (23)	9.4 years	19.5 years	29.8 years
			IQR^●^ 4.9–13.8	IQR^●^ 15.5–25.0	IQR^●^ 21.4–35.7
		F 15 (65%)	10.5 years	19.9 years	29.2 years
			IQR^●^ 6.5–14.7	IQR^●^ 12.4–25.3	IQR^●^ 19.5–36.8
		M 8 (35%)	6.9 years	19.1 years	30.9 years
			IQR^●^ 4.4–11.7	IQR^●^ 18.4–22.4	IQR^●^ 24–33.4

^a^ Subsequent thyroid carcinoma.

^b^ Subsequent thyroid adenoma.

^●^ IQR, interquartile range – lower–upper quartile.

The median age at first cancer diagnosis was 9.8 years (IQR: 4.1–14.6) in the STC group and 9.4 years (IQR: 4.9–13.8) in the STA group. The most commonly administered chemotherapeutic agents for primary cancer treatment were alkylating agents, antitumor antibiotics, and topoisomerase inhibitors. Radiotherapy involving thyroid exposure was administered to 26 patients (59.1%) in the STC group and 19 patients (82.6%) in the STA group. Although radiotherapy involving thyroid exposure was more common in the STA group, the difference did not reach statistical significance (Fisher’s exact test, *P* = 0.06, OR = 3.23, 95% CI: 0.87–15.28). Among irradiated patients, the median radiation dose was 29.1 Gy (IQR: 25.0–30.4) in the STC group and 30.0 Gy (IQR: 24.7–38.0) in the STA group.

Statistical evaluation revealed no significant differences between the STC and STA groups regarding these treatment factors. A comparison of treatment factors between the STC and STA groups is presented in Table 2.

**Table 2:** First cancer treatment in patients with STC and STA.

First cancer diagnosis	STC^a^/STA^b^	RT neck	Other RT regions thyroid exposure	Alkylating agents	Antitumor antibiotics	Antimetabolites	Platinum-based agents	Topoisomerase and mitotic inhibitors
Hodgkin lymphoma	21/13	4/5	16/8	17/11	9/6	3/1	0/0	21/13
Neuroblastoma	7/0	0/0	2/0	7/0	5/0	0/0	4/0	7/0
CNS tumors	4/3	0/0	3/2	2/3	0/0	1/0	1/3	1/3
Non-Hodgkin lymphoma	4/4	1/2	0/1	4/4	3/3	3/2	0/0	4/4
Germ cell tumors	4/0	0/0	0/0	1/0	2/0	0/0	2/0	1/0
Ewing sarcoma	2/0	0/0	0/0	2/0	2/0	0/0	0/0	2/0
Nephroblastoma	1/0	0/0	0/0	0/0	1/0	0/0	0/0	1/0
NET*^A^*	1/0	0/0	0/0	0/0	0/0	0/0	0/0	0/0
Osteosarcoma	0/1	0/0	0/0	0/1	0/1	0/1	0/1	0/0
Retinoblastoma	0/1	0/0	0/0	0/1	0/0	0/0	0/0	0/1
Histiocytosis	0/1	0/1	0/0	0/1	0/1	0/1	0/1	0/1
Total	44/23	5/8	21*/11**^#^**	33/21	22/11	7/5	7/5	37/22

^a^ Subsequent thyroid cancer.

^b^ Subsequent thyroid adenoma.

* Sixteen patients received irradiation of the upper lymphatic system, three underwent craniospinal axis irradiation, and two received total body irradiation.

^#^ Nine patients received irradiation of the upper lymphatic system, and two underwent craniospinal axis irradiation.

^A^ NET, neuroendocrine tumor (genetic testing was negative).

1b) The incidence of STCs was higher among survivors than in the age- and sex-matched national general population, with a 7.6-fold increase in women and a 23.6-fold increase in men (Table 3). Although the absolute incidence was higher among female survivors, the relative increase was substantially greater among male survivors because of the lower incidence of thyroid cancer among men in the national general population. More detailed information is provided in Tables 3 and 4.

**Table 3:** Standardized incidence ratios (SIRs)^#^ comparing observed and expected incidence rates, with 95% confidence intervals.

Sex	Rate_per_100k_sir^#^	95%_CI_lower_sir^#^	95%_CI_upper_sir^#^
Female	7.63	4.84	11.45
Male	23.61	13.75	37.80

^#^ The SIR indicates how many times higher the observed incidence of thyroid cancer in the study cohort is than the expected incidence in the national general population with the same age, sex, and calendar year distribution.

**Table 4:** Number of observed cases and incidence rates per 100,000 person-years, with 95% exact Poisson confidence intervals.

Sex	Total_count^#^	Rate_per_100k	95%_CI_lower	95%_CI_upper
Female	23	50.48	32.00	75.74
Male	17	36.15	21.06	57.87

^#^ Patients aged ≤40 years at the time of STC diagnosis.

2) Among patients with STCs, additional SMNs were detected in six patients (14%). In addition, 13 SBNs at other anatomical sites were identified in nine patients (20%). One patient had an SMN diagnosed before STC diagnosis, whereas five patients developed SMNs after STC diagnosis. Eleven SBNs were histologically confirmed after STC diagnosis. In the STA cohort, SMNs were present in three patients (13%), including one patient with an SMN diagnosed before STA diagnosis. A total of eight SBNs were identified in six patients (26%), all of which were diagnosed before STA diagnosis. The detailed histopathological characteristics of additional SMNs and SBNs in patients with STCs and STAs are summarized in Table 5. There was no significant difference between the STC and STA groups in the incidence of other SMNs or SBNs.

**Table 5:** Detailed histopathological classification of additional SBNs and SMNs in patients with STC and STA.

	First cancer	Pts. w/multiple SNs	SMNs	SBNs
STC	Hodgkin lymphoma	4	Basal cell carcinoma Melanoma Renal carcinoma	Myoma (2×) Dermatofibroma
	Neuroblastoma	3	Melanoma	Neurinoma Bone fibroma Cystadenoma
	CNS tumor	1	Basal cell carcinoma	Neurinoma
	Nephroblastoma	1	Colorectal carcinoma	Fibroadenoma
	Non-Hodgkin lymphoma	2		Colon polyps Bone cyst Fibroma
	Germ cell tumor, non-CNS	1		Lipoma
	NET*	1		Lipoma
STA	Hodgkin lymphoma	4	Breast carcinoma Colorectal carcinoma Basal cell carcinoma	Fibroadenoma Myoma
	Non-Hodgkin lymphoma	2		Teratoma Meningioma Osteochondroma
	CNS tumor	1		Myxoma Nephroma
	Germ cell tumor	1		Fibroadenoma

SNs, subsequent neoplasms; STC, subsequent thyroid carcinoma; STA, subsequent thyroid adenoma.

SBN, subsequent benign neoplasm; SMN, subsequent malignant neoplasm.

* NET, neuroendocrine tumor (genetic testing was negative).

3) Among patients in our subsequent neoplasms cohort, we identified 143 patients without a genetic syndrome who had survived for at least 5 years after the diagnosis of their first SMN. Additional subsequent neoplasms after the diagnosis of the first SMN were observed in 25 patients (17%). Among patients whose first SMN was STC, additional SMNs occurred in 11.6%, compared with 6% among those whose first SMNs was of another type. This difference did not reach statistical significance. In contrast, the incidence of SBNs was significantly higher in patients with STC as their first SMN than in those with other first SMNs (*P* < 0.05). Detailed data are summarized in Table 6. STC was the most frequent first SMN, occurring in 43 patients (30%). In one additional patient, colorectal carcinoma was the first SMN, and STC occurred as the second SMN. During the follow-up period, a total of 16 subsequent neoplasms were documented in nine patients in the STC group.

**Table 6:** Subsequent neoplasms following SMN1 in childhood cancer survivors from the LCP cohort.

SMN1	No. of pts. with SMN1	≥SMN2	≥SBN1	No. of pts. with multiple SNs
STC	43 (30%)*	5	11	9^#^(21%)
Breast carcinoma	26 (18%)	0	1	1 (4%)
Skin cancer^1^	19 (13%)	1	4	5 (26%)
Leukemia/MDS/lymphoma	12 (8%)	1	0	1 (8%)
Genitourinary tumors^2^	11 (8%)	1	0	1 (9%)
Other tumors^3^	9 (6%)	1	1	2 (22%)
Soft tissue sarcoma^4^	7 (5%)	0	2	2 (29%)
Gastrointestinal carcinomas^5^	6 (4%)	2	0	2 (33%)
CNS tumors^6^	5 (3%)	0	2	2 (40%)
Malignant bone tumors^7^	5 (3%)	0	0	0
Total number	143	11	21	25 (17%)

SMN, subsequent malignant neoplasm; SBN, subsequent benign neoplasm; SNs, subsequent neoplasms; STC, subsequent thyroid carcinoma.

* One STC was SMN2; 43 STCs were first subsequent malignant neoplasms*.*

^#^ Seven patients had ≥ SMN2 and/or ≥ SBN1.

^1^ Number of patients with skin cancer: basal cell carcinoma, 16; melanoma, 2; squamous cell carcinoma of the skin, 1.

^2^ Number of patients with genitourinary tumors: renal carcinoma, 4; bladder carcinoma, 2; prostate carcinoma, 1; nephroblastoma, 1; ovarian carcinoma, 1; uterine carcinoma, 2.

^3^ Number of patients with other tumors: tongue carcinoma, 2; retroperitoneal germ cell tumor, 2; carcinoid tumor, 1; lung carcinoma, 1; parotid gland carcinoma, 1; neuroblastoma, 1; carcinoma of unknown origin, 1.

^4^ Number of patients with soft tissue sarcoma: bladder leiomyosarcoma,4; ovarian leiomyosarcoma,1; undifferentiated sarcoma,1; orbital sarcoma, 1.

^5^ Number of patients with gastrointestinal carcinomas: intestinal adenocarcinoma, 4; rectal adenocarcinoma, 1; esophageal adenocarcinoma, 1.

^6^ Number of patients with CNS tumors: low-grade glioma, 2; medulloblastoma, 1; astrocytoma, 1; high-grade glioma, 1.

^7^ Number of patients with malignant bone tumors: osteosarcoma, 3; chondrosarcoma, 1; Ewing sarcoma, 1.

In the same cohort, we identified 157 patients without a genetic syndrome who had survived for at least 5 years after the diagnosis of their first SBN. Additional tumors diagnosed after the first SBN were observed in 39 patients (25%). Detailed data are summarized in Table 7. Benign epithelial tumors were the most common SBNs, with STA being the most frequent subtype, accounting for 17 cases (31%) among 54 patients with epithelial benign tumors.

**Table 7:** Subsequent neoplasms following SBN1 in childhood cancer survivors from the LCP cohort.

SBN1	No. of pts. with SBN1	≥ SMN 1	≥ SBN 2	No. of pts. with multiple SNs
STA	17 (11%)*	2	0	2 (12%)
Mesenchymal^1^	41 (26%)	5	12	14^#^(34%)
Other epithelial^2^	37 (24%)	5	3	7^#^(19%)
Meningioma	28 (18%)	4	3	6^#^(21%)
Benign bone lesions^3^	16 (10%)	3	3	6 (38%)
Other benign tumors^4^	11 (7%)	0	4	4 (36%)
Benign neural tumors^5^	7 (4%)	0	0	0
Total number	157	19	25	39 (25%)

SBN, subsequent benign neoplasm; SMN, subsequent malignant neoplasm; SNs, subsequent neoplasms; STA, subsequent thyroid adenoma.

* In five survivors, STA was SBN2 and in one survivor, it was SBN3.

^#^ Five patients had ≥SMN1 and/or ≥SBN2 in total.

^1^ Number of patients with mesenchymal SBN1: myomas, 13; lipomas, 12; fibromas/fibrolipoma, 7; leiomyomas, 3; angiomyolipomas, 1; lymphangiomas/hemangiomas, 3; myofibromatosis, 1; myxoma, 1.

^2^ Number of patients with other epithelial SBN1: breast adenoma/fibroadenoma, 13; polyps, 5; parotid adenoma, 3; liver adenoma, 3; papilloma, 4; parathyroid adenoma, 2; pituitary adenoma, 2; submandibular adenoma, 1; renal oncocytoma, 1; urethral adenoma, 1; ovarian adenoma, 1; thyroid adenoma, 1.

^3^ Number of patients with benign bone lesions: osteochondroma, 5; enchondroma, 3; non-ossifying fibroma (NOF), 3; osteoma, 2; aneurysmal bone cyst, 1; chondroblastoma, 1; osteoclastoma, 1.

^4^ Number of patients with other benign tumors: teratoma, 3; histiocytoma, 2; dermoid cyst,2; pheochromocytoma, 1; insulinoma, 1; hamartoma, 1; serous borderline tumor of ovary,1.

^5^ Number of patients with benign neural tumors: schwannoma, 2; ganglioglioma, 3; ganglioneuroma, 1; oligodendroglioma, 1.

## Discussion

In this study, we compared the occurrence of STCs and STAs in childhood cancer survivors, focusing on treatment-related risk factors and their associations with the development of other subsequent neoplasms. To our knowledge, data on these associations outside the context of known genetic syndromes remain limited.

In agreement with other studies of survivors, we observed that STC is one of the most common SMNs after childhood cancer treatment (1, 2, 3, 25). In our cohort, the prevalence of STC was 1%, which is consistent with previous reports (27, 28). Patients with STA accounted for only 0.5% of the study population; however, the true incidence is likely to be higher because STA was assessed only in survivors who had undergone total thyroidectomy. Children are more susceptible to SMNs, likely because developing tissues are more sensitive to chemotherapeutic agents and, in particular, to radiation exposure (29). This observation underscores the well-recognized balance in radiation oncology between therapeutic benefit and the potential risks associated with radiation exposure to both tumors and surrounding normal tissues (1, 9, 30, 31). Experimental animal studies suggest increased susceptibility to radiation-induced thyroid cancer at younger ages, possibly related to a higher proportion of radiosensitive cells, including immature thyrocytes with stem-like characteristics that may persist following irradiation (32).

Consistent with these observations, a substantial proportion of patients in our cohort had previously undergone radiotherapy, including 59% of patients with STC and 83% of those with STA. Established risk factors for the development of STC and STA include direct neck irradiation, mantle field radiotherapy, and craniospinal irradiation (33). The exact dose delivered to the thyroid gland is difficult to determine because of the variety of irradiation techniques employed over the long follow-up period. The median radiotherapy dose to the neck region was 29.1 Gy for STC and 30 Gy for STA, although the actual dose received by the thyroid gland was likely lower. For example, in nine patients who received mantle field irradiation for Hodgkin lymphoma, phantom models simulating the commonly used 40 Gy mantle field suggest that the thyroid gland could receive a minimum dose of 12 Gy (34).

Radiotherapy also appeared to be a contributing risk factor for the development of additional SMNs in patients with subsequent thyroid neoplasms. Notably, seven of the nine patients (78%) developed SMNs within the previously irradiated field.

In both the STC and STA groups, alkylating agents were the most frequently administered chemotherapeutic agents. Previous studies have reported an association between exposure to these drugs and the development of SMNs (35, 36). Alkylating agents administered without radiotherapy to the thyroid gland have been shown to increase the risk of STC, whereas this effect appears to be attenuated when chemotherapy is combined with radiotherapy (27). In our cohort, only two patients with STA had not received alkylating agents but had undergone neck radiotherapy. Among patients with STC, 11 had not been treated with alkylating agents; however, only six of them had not received neck radiotherapy.

As anticipated, the highest incidence of STC and STA was observed after treatment for Hodgkin lymphoma. The main risk factors were radiotherapy to the neck and upper lymphatic regions and chemotherapy with alkylating agents. These findings are consistent with previous reports showing the highest cumulative incidence of SMNs among Hodgkin lymphoma survivors, with STC being a well-recognized late effect (2, 3, 36, 37).

Females are reported to be at a higher risk of developing thyroid cancer after radiation exposure (10, 11, 28, 37, 38). In agreement with these data, females in our cohort were more frequently represented than males with both types of thyroid neoplasms: 59% for STC and 65% for STA. Although women were more frequently represented among patients with subsequent thyroid neoplasms than in the cohort of patients with primary neoplasms, the association was only of borderline statistical significance, indicating that the observed difference should be interpreted with caution. This finding is consistent with more recent studies suggesting that the risk of STC may be independent of sex, contrary to earlier reports (6). The median age at STC diagnosis was 26.4 years in females and 23.3 years in males, which is consistent with previous findings (27). STC occurs at a substantially younger age than thyroid cancer in the general population. According to data from the Institute of Health Information and Statistics, the median age at diagnosis of primary thyroid cancer is 51 years in women and 56 years in men. The younger age at STC diagnosis compared with thyroid cancer in the general population may have been partly influenced by regular ultrasound surveillance in survivors, leading to earlier detection (surveillance bias). This limitation should therefore be considered when interpreting the age difference. However, we assume that surveillance alone is unlikely to explain the substantially younger age at diagnosis. The median age at STA diagnosis was 29.8 years, slightly higher than that at STC diagnosis. This older age at SBN diagnosis is consistent with previous findings (22), which reported the highest cumulative incidence of benign tumors at approximately 32 years of age among survivors treated with radiotherapy.

Children younger than 5–10 years are particularly sensitive to the carcinogenic effects of ionizing radiation (33), which is consistent with our findings: the median age at primary cancer diagnosis was 9.8 years in patients with STC and 9.4 years in those with STA. Previous studies reported a median latency of 19–20 years for STC following treatment of the first primary cancer (33). However, the median latency in our cohort was 15.8 years, likely reflecting earlier detection through regular surveillance of asymptomatic patients and diagnosis of STC at earlier stages.

Papillary carcinoma was the most common histopathological type of STC (86%), consistent with findings from previous studies (39, 40, 41). Furthermore, it is the most common type of thyroid cancer in the general population (42). Similarly, follicular adenoma (52%) was the most common histopathological type of STA, in agreement with earlier reports (15, 43, 44).

The increased incidence of other SBNs and SMNs in patients with STC and STA may indicate increased susceptibility to tumorigenesis. The occurrence of additional subsequent neoplasms is well documented in hereditary cancer syndromes, including multiple endocrine neoplasia type 2, PTEN hamartoma tumor syndrome, familial adenomatous polyposis, DICER1 syndrome, and Li–Fraumeni syndrome. However, data on additional subsequent neoplasms in patients without identifiable genetic syndromes remain limited. We assume that the development of multiple subsequent neoplasms in patients with STC and STA may be influenced by a combination of primary cancer therapy and polygenic risk (25).

Seven of 67 patients (10%) developed other SMNs after subsequent thyroid neoplasms, supporting previous observations that survivors remain at risk of developing multiple subsequent neoplasms (19, 21). In another study, 11% of patients with SBNs also had SMNs (22), compared with 12% of patients who developed SMNs after STA in our cohort. Although STA can also serve as a precursor to STC (44), total thyroidectomy in all patients with STA precluded evaluation of progression to carcinoma. Only one patient in our cohort developed STA in the left lobe and STC in the right lobe.

The risk of developing subsequent neoplasms in survivors has been reported to be approximately sixfold higher than that in the general population (30). Notably, the prevalence ratio for STC reached 43.5 in a cohort of more than 11,000 childhood cancer survivors (45). This risk is further amplified for radiotherapy-associated SMNs; radiotherapy has been shown to increase the incidence of STC by 15- to 53-fold (14). Consistent with these findings, female survivors of reproductive age have been reported to have an approximately 16-fold higher risk of STC than the general population (46). A similarly marked increase was observed in our cohort, with the incidence of STC being approximately 7.6-fold higher in women and 23.6-fold higher in men than in the age- and sex-matched national general population.

Given this increased risk, regular screening of survivors may facilitate the early detection of STC and could potentially contribute to improved long-term outcomes. Only two patients died, both from causes unrelated to thyroid malignancy (cardiotoxicity and COVID-19-related multiorgan failure).

Based on the recommendations of the International Guideline Harmonization Group and the PanCare guidelines, thyroid ultrasound screening is recommended to begin 5 years after the initiation of radiotherapy (47, 48, 49). At our center, thyroid monitoring is also typically initiated 5 years after radiotherapy.

It is important to note that increased testing for STC may induce anxiety, even when benign changes are identified (50). As previously demonstrated, current radiotherapy and chemotherapy approaches have reduced the risk of SMNs; however, this risk persists (51, 52, 53). It can therefore be assumed that the incidence of STC may also decrease over time.

### Limitations

Although treatment-related risk factors are well established, the extent to which genetic cancer susceptibility modifies treatment-related risks for developing subsequent thyroid neoplasms remains unclear. All patients with diagnosed genetic syndromes were excluded; however, undiagnosed cases may have been present in the cohort. Clinically silent STA may have remained undetected in survivors who did not meet the criteria for regular thyroid ultrasound surveillance. For most SMNs, irrespective of treatment factors, a so-called polygenic risk is assumed, largely influenced by lifestyle factors (e.g. smoking, poor diet, sedentary lifestyle, inadequate sun protection, overweight, and drug or alcohol abuse). These factors were not considered in relation to the risk of subsequent thyroid neoplasms.

### Strengths

Long-term follow-up of childhood cancer survivors at our institution provided detailed information not only on the incidence of STC and STA but also on the occurrence of other subsequent neoplasms.

## Conclusion

Among patients with subsequent thyroid neoplasms, STC predominated over STA. Neck radiotherapy emerged as a major treatment-related risk factor for both entities. Female sex and a history of Hodgkin lymphoma were common in both groups. Although the age at primary cancer diagnosis was similar, STC was generally diagnosed at a younger age. Compared with the national general population, STC occurs more frequently in survivors of both sexes and at a younger age. A substantial proportion of patients with subsequent thyroid neoplasms developed additional neoplasms, highlighting the need for long-term, comprehensive oncological surveillance in this population.

## Declaration of interest

The authors declare that there is no conflict of interest that could be perceived as prejudicing the impartiality of this work.

## Funding

This study was co-funded by the EU and the state budget of Czechia through the OP JAC, project SALVAGE (No. CZ.02.01.01/00/22_008/0004644).

## Ethics statement

Ethical approval for this study was waived by the Ethics Committee of Motol and Homolka University Hospital because of the retrospective nature of the study and because all procedures were performed as part of routine care.

## AI disclosure

The authors did not use generative AI for content creation. AI tools were used solely for language editing, together with DeepL Translator, and all scientific content remains the responsibility of the authors.
